# Genetic toolbox for controlled expression of functional proteins in *Geobacillus* spp.

**DOI:** 10.1371/journal.pone.0171313

**Published:** 2017-02-02

**Authors:** Ivan Pogrebnyakov, Christian Bille Jendresen, Alex Toftgaard Nielsen

**Affiliations:** The Novo Nordisk Foundation Center for Biosustainability, Technical University of Denmark, Kgs. Lyngby, Denmark; Virginia Commonwealth University, UNITED STATES

## Abstract

Species of genus *Geobacillus* are thermophilic bacteria and play an ever increasing role as hosts for biotechnological applications both in academia and industry. Here we screened a number of *Geobacillus* strains to determine which industrially relevant carbon sources they can utilize. One of the strains, *G*. *thermoglucosidasius* C56-YS93, was then chosen to develop a toolbox for controlled gene expression over a wide range of levels. It includes a library of semi-synthetic constitutive promoters (76-fold difference in expression levels) and an inducible promoter from the *xylA* gene. A library of synthetic *in silico* designed ribosome binding sites was also created for further tuning of translation. The P_*xylA*_ was further used to successfully express native and heterologous xylanases in *G*. *thermoglucosidasius*. This toolbox enables fine-tuning of gene expression in *Geobacillus* species for metabolic engineering approaches in production of biochemicals and heterologous proteins.

## Introduction

For decades, thermophilic bacteria have been used in biotechnology. Their applications have mainly been confined to their thermostable enzymes, one of the most prominent examples being the *Taq* polymerase from *Thermus aquaticus* [1]. The global market for industrial enzymes in 2015 was estimated to be US$ 4.4 billion [2] with thermostable enzymes playing an ever increasing role [3]. The genus *Geobacillus*, comprising some thermophilic species previously belonging to genus *Bacillus* [4], has also been used in this regard. Examples of industrially relevant enzymes isolated from *Geobacillus* species include proteases [5], amylases [6], lipases, [7] and xylanases [8], to mention just a few.

Recently, however, there has been a growing interest in thermophiles as biotechnological hosts [9]. Thermophilic species of Bacteria and Archaea are promising candidates for a number of applications, from production of chemicals [10]; [11]; [12] to extraction of metals from mineral ores [13]. *Geobacillus* species are also keeping up with this trend. For example, successful metabolic engineering of ethanol [14]; [15] and isobutanol [16] production in *G*. *thermoglucosidasius* was achieved. Some *Geobacillus* strains have also been used for heterologous protein expression. A protein from an archaeon, which was insoluble when expressed in *Escherichia coli*, was successfully folded in *G*. *kaustophilus* [17]. This illustrates an important feature: some thermostable proteins may need thermophilic rather than mesophilic hosts for proper expression. Apart from that, thermophilic bacteria may offer other interesting advantages over commonly used hosts, like *E*. *coli* and *B*. *subtilis*. These include higher reaction rates at elevated temperatures, decreased risk of contamination with mesophilic organisms, as well as ease of recovery of volatile compounds, such as ethanol [18]. In addition to that, *Geobacillus* species can utilize a wider arrange of carbon sources, notably C5 sugars like xylose and arabinose, and their polymers, xylan and arabinan [19]. These polymers are the most abundant parts of hemicellulose, which constitutes a considerable fraction of plant biomass [20]. Strains that are able to degrade cellulose have also been reported [21]. All these properties point to a high potential of geobacilli for biotechnology, e.g. for plant biomass conversion into value-added chemicals. Some strains have already been employed by commercial companies, e.g. TMO Renewables (www.tmo-group.com), ReBio Technologies (www.rebio.co.uk) and C5-6 Technologies (www.c56technologies.com).

A number of tools for genetic engineering of *Geobacillus* spp. have been developed, including transformation techniques [22]; [23]; episomal [24]; [25] and integration [14] vectors; selection markers [26]. A more detailed overview of current tools for engineering of *Geobacillus* strains can be found in [18].

Although metabolic engineering and heterologous protein expression has been achieved in *Geobacillus*, there have been few attempts to systematically characterize genetic parts, i.e. promoters and ribosome binding sites (RBS) for these species [18]; [27]. Production of biochemicals through metabolic engineering often requires the expression of a number of pathway enzymes, and the best production yields are not always achieved by the highest amount of the target pathway enzymes in the cell, but rather by fine-tuning of the expression levels of the individual enzymes [28]. Similarly, expression levels of heterologous proteins sometimes should be experimentally adjusted for optimal yields [29]. To achieve this goal, a set of both constitutive and inducible promoters as well as RBS’s of various strengths are needed. Recently, Reeve et al. described construction of a library of promoters using mutagenic PCR of a parent strong promoter from G. thermoglucosidasius [30]. The mutation rate in this method was approximately 10%, which makes the resulting promoters highly similar. This may cause homologous recombination between the promoters from this library and their wild type parent on the chromosome.

In this study we have generated a toolbox for controlled gene expression in *G*. *thermoglucosidasius*. The toolbox includes libraries of promoters and RBS sequences of various strengths that were screened using the superfolder green fluorescent protein (sfGFP) as a reporter. Importantly, much of the promoters’ sequences were randomized, so that they have low similarity. Additionally, a xylose-inducible promoter that enables strong and titratable expression was characterized. The developed tools were used to demonstrate the successful expression of two xylanases from *Geobacillus* species.

## Materials and methods

### Strains, plasmids and media

Bacterial strains and plasmids used in this study are listed in Table 1. *E*. *coli* cells were grown in lysogeny broth (LB) [30] with 100 μg/mL ampicillin added when needed for selection of plasmids. *Geobacillus* strains were grown in either of several media. The mTGP (modified from [25]) medium contained per liter: 17 g tryptone, 3 g soy peptone, 5 g NaCl, 2.5 g K_2_HPO_4_. After autoclaving, sterile solutions were added to final concentrations: 4 mL/L glycerol, 4 g/L sodium pyruvate, 0.59 mM MgSO_4_, 0.91 mM CaCl_2_ and 0.04 mM FeSO_4_; agar to 1.5% (w/v) was added to solidify the medium when needed. Thermophile minimal medium (TMM) was adapted from [31] with some modifications. It contained, per liter: Six salts solution (SSS), 930 mL; 1 M MOPS (pH 8.2), 40 mL; 1 mM FeSO_4_ in 0.4 M tricine, 10 mL; 0.132 M K_2_HPO_4_, 10 mL; 0.953 M NH_4_Cl, 10 mL; 1 M CaCl_2_, 0.5 mL; trace elements solution, 0.5 ml; Wolfe’s vitamin solution, 10 mL. SSS contained, per 930 mL: 4.6 g NaCl, 1.35 g Na_2_SO4, 0.23 g KCl, 0.037 g KBr, 1.72 g MgCl_2_·6H_2_O, 0.83 g NaNO_3_. Trace elements solution contained, per liter: 1 g FeCl_3_·6H_2_O, 0.18 g ZnSO_4_·7H_2_O, 0.12 g CuCl_2_·2H_2_O, 0.12 g MnSO_4_·H_2_O, 0.18 g CoCl_2_·6H_2_O. Yeast extract in final concentrations of 0.02% (w/v) or 0.05% (w/v) was added when indicated. For *Geobacillus* spp. selections were done using kanamycin at a final concentration of 12.5 μg/mL.

**Table 1:** Strains and plasmids used in this study.

Strain or plasmid	Description	Reference
**Strains**		
*E*. *coli* NEB5-alpha	*fhuA2 Δ(argF-lacZ)U169 phoA glnV44 Φ80 Δ(lacZ)M15 gyrA96 recA1 relA1 endA1 thi-1 hsdR17*	New England Biolabs
*G*. *thermoglucosidasius* C56-YS93	Hot spring isolate of *G*. *thermoglucosidasius*	[32]
**Plasmids**		
pUCG18	pMB1 and pBST22 *ori*; Amp^R^, Km^R^ *E*. *coli*-*Geobacillus* shuttle vector	[25]
pIPGE	pMB1 and pBST22 *ori*; Amp^R^, Km^R^; P_*groES*_::sfGFP with P_*groES*_ having the CIRCE sequence deleted	This study
pIP1	Modified pIPGE with P_*groES*_ derivative P1	This study
pIP2	Modified pIPGE with P_*groES*_ derivative P2	This study
pIP3	Modified pIPGE with P_*groES*_ derivative P3	This study
pIP4	Modified pIPGE with P_*groES*_ derivative P4	This study
pIP5	Modified pIPGE with P_*groES*_ derivative P5	This study
pIP6	Modified pIPGE with P_*groES*_ derivative P6	This study
pIP7	Modified pIPGE with P_*groES*_ derivative P7	This study
pIP8	Modified pIPGE with P_*groES*_ derivative P8	This study
pIP9	Modified pIPGE with P_*groES*_ derivative P9	This study
pIP0	Modified pIPGE with P_*groES*_ derivative P10	This study
pIP11	Modified pIPGE with P_*groES*_ derivative P11	This study
pIP12	Modified pIPGE with P_*groES*_ derivative P12	This study
pIP13	Modified pIPGE with P_*groES*_ derivative P13	This study
pIP14	Modified pIPGE with P_*groES*_ derivative P14	This study
pIP15	Modified pIPGE with P_*groES*_ derivative P15	This study
pIP16	Modified pIPGE with P_*groES*_ derivative P16	This study
pIP17	Modified pIPGE with P_*groES*_ derivative P17	This study
pIP18	pMB1 and pBST22 *ori*; Amp^R^, Km^R^; P_*pfl*_::sfGFP	This study
pIP19	Derivative of pIPRL with RBS replaced with R1	This study
pIP20	Derivative of pIPRL with RBS replaced with R2	This study
pIP21	Derivative of pIPRL with RBS replaced with R3	This study
pIP22	Derivative of pIPRL with RBS replaced with R4	This study
pIP23	Derivative of pIPRL with RBS replaced with R5	This study
pIP24	Derivative of pIPRL with RBS replaced with R6	This study
pIP25	pMB1 and pBST22 *ori*; Amp^R^, Km^R^; P_*xylA*_::sfGFP	This study
pIP26	pMB1 and pBST22 *ori*; Amp^R^, Km^R^; *xylR*, P_*xylA*_::sfGFP	This study
pIP27	pMB1 and pBST22 *ori*; Amp^R^, Km^R^; P_*xylA*_::Geoth_2264	This study
pIP28	pMB1 and pBST22 *ori*; Amp^R^, Km^R^; P_*xylA*_::Gtng_1761	This study

### DNA manipulations

Genomic DNA was extracted using the Wizard^®^ Genomic DNA Purification Kit (Promega) according to producer’s specifications. Plasmid extractions were performed using NucleoSpin^®^ Plasmid EasyPure kit (Macherey-Nagel).

### PCR and cloning

Primers used in this study are listed in Table 2. PCR of DNA fragments for USER cloning was performed with primers containing uracil using the Phusion U Hot Start DNA Polymerase (Thermo Fisher Scientific). Colony PCR was performed with *Taq* 2x Master Mix (New England Biolabs) in order to detect positive colonies. Reactions were done according to manufacturers’ recommendations with elongation times and annealing temperatures adjusted for specific targets and primers. In most cases annealing temperature was 60°C and elongation time was programmed at 30 seconds per 1 kb. DNA cloning was performed using USER (uracil-specific excision reagent) technology. It is a simple and robust method, allowing seamless DNA insertions [33]. PCR-amplified DNA fragments containing a primer-incorporated uracil close to both of their 5’-ends were mixed (purification after PCR was not necessary) and treated with *Dpn*I enzyme (Thermo Fisher Scientific) for 30 min at 37°C to digest template DNA. USER^™^ enzyme (New England Biolabs) was then added, and the mixture was incubated in three steps: 1) 37°C for 15 min; 2) 12°C for 15 min; 3) 10°C for 10 min. It was then transferred on ice and mixed with chemically competent *E*. *coli* cells.

**Table 2:** Oligonucleotides used in this study.

Name	Sequence 5’ → 3’	Target
PNJ24b	AATTCGUAATCATGGTCATAGCTGTTTCC	sfGFP with pUCG18 backbone
PNJ94	ATGAGUAAAGGCGAAGAGCTG	
PNJ97	ACGAATUCCATCATCTAATTCATATTGTTCAACATTTCAC	P_*pfl*_
PNJ98	ACTCAUAACAGTTTCCCTCCCATGCATC	
PNJ205	ATCTGUTTATATAACAGATTTGTAAAAATGTATATAACAGC	
PNJ207	ACAGAUGACGTACACGCCGAAGGAAAGGGCCCATGAGTAAAGGCGAAGAGC	P_*pfl*_ with RBS207
PNJ210	ACAGAUATTTAAAAAACAAGAGGGGTAACATGAGTAAAGGCGAAGAGC	P_*pfl*_ with RBS210
PNJ212	ACAGAUTTCACAAGTAACAAAGGGGAAGAGGGGTAACATATGAGTAAAGGCGAAGAGC	P_*pfl*_ with RBS212
PNJ213	ACAGAUTGTACACACAAGAGGAGGGAGTATTATTATGAGTAAAGGCGAAGAGC	P_*pfl*_ with RBS213
PNJ215	ACAGAUGATATATCACAAAGGAGGGTAACAACATGAGTAAAGGCGAAGAGC	P_*pfl*_ with RBS215
PNJ217	ACAGAUCCCACTACACAAGAACAAGAAGGAGGATTATATATGAGTAAAGGCGAAGAGC	P_*pfl*_ with RBS217
PNJ267	ACGAATUCGGCAAAACAACCGGCTCCTTTTGCTC	*groES* promoter with CIRCE deleted
PNJ268	ACGATAGUTTTCGCCGTTCTTACACACTTATAATATTAATGAACTTCTTTCCGTTTTGC	
PNJ269	ACTATCGUTAAGGAGGTCGTTTCCCATGAGTAAAGGCGAAGAGCTGTTCAC	
PNJ388	ACACACUWWWWATATTAWWN_15_TTGCAANWWNNWWWTGCAAAAAAATAACTGTTTTTCTCTCCTAAAGAAGAAAG	*groES* promoter with randomized sequences
PNJ389	AGTGTGUAAGAACGGCGAAAACTATCGTTAAG	
PNJ458	AGGGGGAUCTAGACATGTTGAAAAGATCGCGAAAAGCG	GTNG_1761
PNJ459	AAAGCCUCACTTATGATCGATAATAGCCCAATACGCAGG	
PNJ474	AGGGGGAUCTAGACATGAGCAGCTCGCTTCCTTCTCTC	Geoth_2264
PNJ475	AAAGCCUTTACCCGTTGACGACCCTCCAAAAAGC	
PNJ450	ACTCAUATCTAACTCCTCCTTAACTTTTAGTAGATTGTC	P_*xylA*_
PNJ550	ATGGCTCUGGGCAAAATAACTAAGCG	
PNJ551	AGAGCCAUGACAAAAAGAAAGATGGAAGCCATCC	*xylR* incl. promoter and terminator
PNJ552	ACGAATUCATGAAACAGAAACAGTGATTCATTTTTATGTTTGC	

### Transformation of *E*. *coli* and *G*. *thermoglucosidasius*

The procedure was based on the protocol described by [25] with some steps modified. *G*. *thermoglucosidasius* was grown overnight on an mTGP agar plate at 60°C. A single colony was inoculated into 50 mL of pre-warmed liquid mTGP in a 250 ml flask and incubated at 60°C and 250 rpm until the culture reached OD_600_ of 1.6–2. Cells were cooled down on ice for 10 min and harvested by centrifugation at 4000 *g* for 10 min. They were washed three times (4000 *g* for 10 min) with freshly prepared ice-cold electroporation buffer. The buffer contained, per 100 mL: 17.12 g sucrose, 0.042 g MgCl_2_·6H_2_O, 5 mL glycerol. After the last washing step, the cell pellet was suspended in 1 mL of electroporation buffer, distributed in 60 μL aliquots and stored at -80°C until further use.

For the transformation, an aliquot was thawed on ice and mixed with DNA. It was transferred into an electroporation cuvette with a 2 mm gap between electrodes and subjected to a discharge at 2 kV, with a typical time constants of 4–5 ms using the MicroPulser^™^ (Bio-Rad). Cells were dissolved in 3 mL mTGP and recovered at 52°C for 2 hours at 200 rpm. Afterwards they were spun down and seeded on selective agar media plates. Transfromation efficiencies typically were 10^1^−10^2^ colonies per microgram of DNA.

### sfGFP measurement

The sfGFP [34] was used as a reporter to assess the expression levels. It was previously shown to be active in *Geobacillus* species [27]. For quantification of sfGFP expression driven by different promoters and RBS’s, *Geobacillus* strains carrying the respective constructs were grown overnight at 60°C in TMM with 0.05% yeast extract and 0.2% glucose. 2 μL of these cultures were inoculated into 100 μL of fresh pre-heated media in flat-bottom 96-well microtiter plate (Greiner Bio-One) and sealed airtight with VIEWSeal (In Vitro) to prevent water evaporation. Plates were incubated at 60°C and 200 rpm. Periodically fluorescence was measured with the ELx808^™^ Microplate Reader (BioTek) with the excitation at 485 nm and emission at 535 nm. Values at the middle of log phase were taken for analysis. Fluorescence was normalized to OD_600_ measured at the same time.

### Xylanase assay

Xylanase activity was measured with EnzChek^®^ Ultra Xylanase Assay Kit (Life Technologies) according to manufacturer’s instructions. Briefly, cells were grown for 21 hours, reaching similar densities, harvested and lysed with CelLytic^™^ B Plus Kit (Sigma-Aldrich). Cell lysate and supernatant from cultures were diluted and 50 μL of dilutions were mixed with 50 μL of xylanase substrate working solution in flat-bottom 96-well microtiter plate (Greiner Bio-One). They were incubated at room temperature for 40 min and the release of reaction products was measured with the ELx808^™^ Microplate Reader (BioTek) with the excitation at 360 nm and emission at 460 nm. Total protein content was measured with Novagen^®^ BCA Protein Assay Kit (Merck) and xylanase activity was normalized to it.

## Results

### Growth of *Geobacillus* strains on various carbon sources

**Fig 1:**
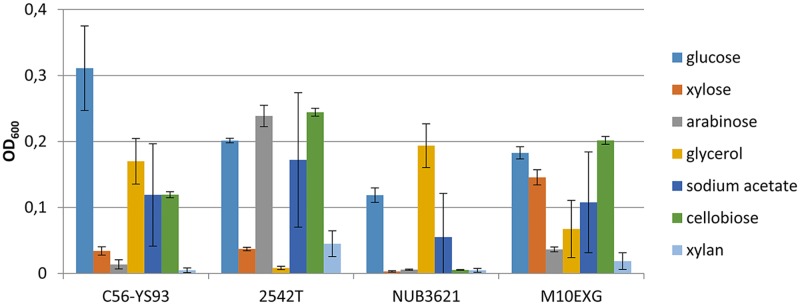
Growth yields. Growth yields of several *Geobacillus* strains in minimal medium containing 0.2% (w/v) of various carbon sources.

In order to assess biotechnological potential of *Geobacillus* spp. we analyzed the ability of four strains to utilize a number of carbon sources: *G*. *thermoglucosidasius* 2542^T^ which was previously used in metabolic engineering of isobutanol production [16]. *G*. *thermoglucosidasius* M10EXG is a natural isolate with high tolerance to ethanol and which is thus a promising host for its production [31]. *G*. *thermoglucosidasius* C56-YS93 is another strain of the same species which genome is sequenced and annotated [32]. *G*. *stearothermophilus* NUB3621 has been used in a number of applications [35] and its genome has been also recently sequenced [27]. Bacterial cultures were grown in minimal medium (TMM) supplemented with a number of different carbon sources. These included sugars (glucose, xylose, arabinose) and more complex carbohydrates (cellobiose and xylan), which constitute a major part of lignocellulosic biomass. Glycerol and acetate were also included in the screening because they are cheap and suitable as industrial carbon sources. Most strains utilized a number of investigated carbon sources, but showed poor growth on xylan (Fig 1). Of these, *G*. *thermoglucosidasius* C56-YS93 had the highest growth yields on glucose combined with good growth on glycerol, acetate and cellobiose. In addition, its genome has been sequenced and annotated and is available online [32], which makes it easier to design and manipulate genetic changes in this strain. Therefore, we chose it for further studies.

### Promoter library

In order to facilitate effective metabolic engineering strategies in *Geobacillus*, it is desirable to have access to a number of promoters with different strengths. A library of semi-synthetic promoters was therefore constructed using a method described by Jensen and Hammer [36]. It includes the randomization of promoter regions between -35 and -10 elements, while leaving these elements intact, as a way to vary promoter strength. This method has been used to construct promoter libraries for *E*. *coli* [37], *Lactococcus lactis* [36], and *Saccharomyces cerevisiae* [38]. Its advantages include the ease of library construction and gradual increments in strength among the resulting promoters [37].

**Fig 2:**
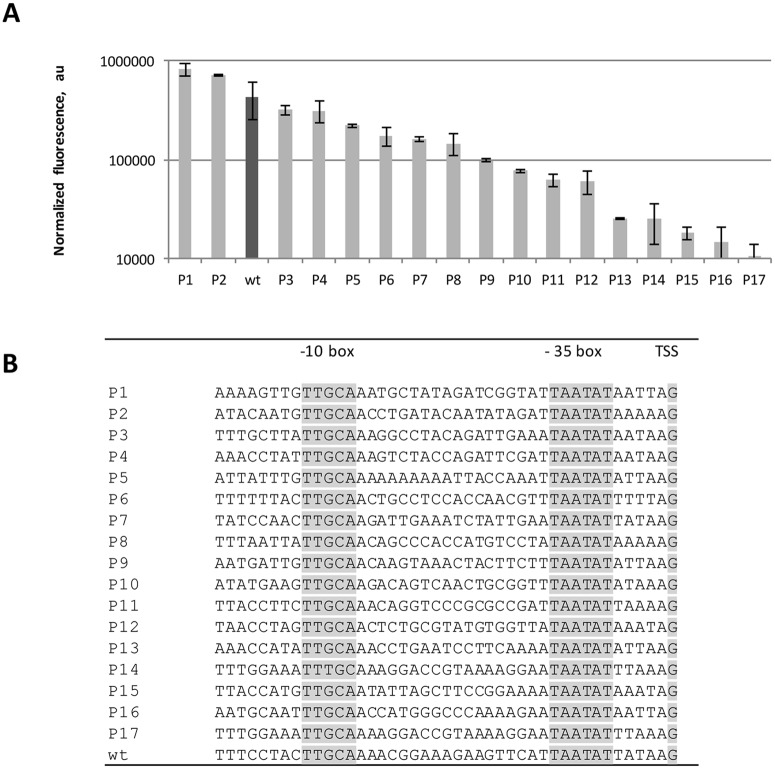
Library of semi-synthetic promoters. (A) Expression levels of sfGFP as measured by fluorescence (arbitrary units, “au”) in the middle of log phase. Parent wild-type (wt) *groES* promoter is marked in dark grey. (B) Sequences of the promoters in the library. The -35 and -10 elements and transcription start site (TSS) are shown in bold.

Here we created a library of synthetic promoters for *Geobacillus* spp, based on the native and strong promoter of the *groESL* operon from *Geobacillus* sp. GHH01 (locus tag GHH_c02820, RefSeq GHH_RS01420). Its regulatory CIRCE sequence [39]; [40] was deleted and the sequences between and around its -35 (TTGCAA) and -10 (TAATAT) elements were randomized using a degenerate oligonucleotide sequence (PNJ388 in Table 1). The ribosome binding site (RBS) was left intact. Fusion of these constructs with sfGFP produced a library that was transformed into *G*. *thermoglucosidasius* C56-YS93. To evaluate the strength of the different promoters, superfolder GFP (sfGFP) fluorescence was measured at the middle of log phase. Low transformation efficiency of *G*. *thermoglucosidasius* limited the library to 17 constructs, which nevertheless covered a 76-fold range of expression levels (Fig 2). Two promoters in the library exhibited higher expression when compared to the *groESL* promoter, while two gave comparable expression levels as to that of the native, and the rest were weaker.

### RBS library

Modulation of translation initiation is often used as a tool to regulate the level of protein production. An array of ribosome binding sites (RBS’s) was therefore constructed using the RBS Calculator [41]; [42]. This software calculates the thermodynamics of interactions between the ribosome and the mRNA. Based on this model, it generates an RBS sequence with a given theoretical translation initiation rate. The model takes into account not only the Shine-Dalgarno sequence, but also sequences flanking it. Since the consensus sequence of bacterial RBS consists of six nucleotides, it is problematic to use RBS Calculator to compare its strength to that of the resulting RBS.

In an alternative randomization approach, Bonde et al. [43] constructed a comprehensive library of almost all possible permutations of six nucleotides acting as RBS (the consensus sequence in *E*. *coli* being AGGAGG) and studied their effect on protein expression. We hypothesized that for a thermophilic organism like *Geobacillus* sp. it is worthwhile using rational RBS design because several of its strains are available in the database of RBS Calculator. RBS libraries described by Bonde et al. were created for *E*. *coli* and might not work in a different genetic context (and at different temperature) of Gram positive bacteria.

**Fig 3:**
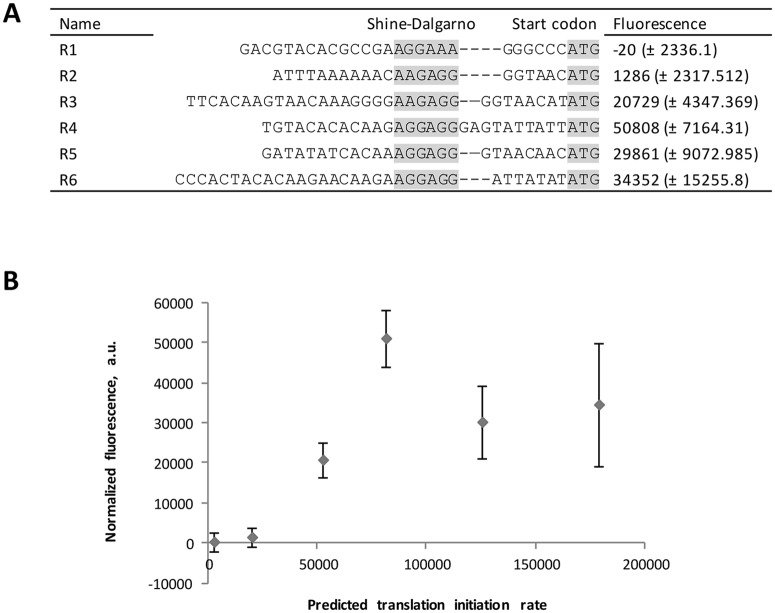
Library of synthetic RBS sequences. The library was constructed using the RBS Calculator and fused to the P_*pfl*_ promoter of *G*. *thermoglucosidasius* C56-YS93 and sfGFP. (A) Sequences of RBS (grey background) and their strengths (standard deviations in parentheses) as measured by sfGFP fluorescence. (B) RBS activity compared to the predicted translation initiation rate. RBS’ on the horizontal axis of the graph appear in the same order as in the table.

A set of RBS’s with a range of different predicted translation initiation rates was created and fused with the promoter P_*pfl*_ of the pyruvate-formate lyase gene (*pfl*) of *G*. *thermoglucosidasius* C56-YS93 (locus tag Geoth_3895, RefSeq GEOTH_RS19245), where the native RBS was replaced by a synthetic one. sfGFP was again used as a reporter for the screening. Two of the tested RBS’s showed low expression levels, while the rest resulted in middle to high expression levels (Fig 3).

### Inducible promoter

Inducible promoters are valuable tools for various applications in molecular biology, because they enable the modulation of gene expression as a function of the concentration of the inducing factor. Here we investigated a xylose-inducible promoter of the xylose isomerase gene (*xylA*), because its homologues in *Bacillus* species have been extensively studied [44]; [45] and used for protein production [46]. The operator sequence of *xylA* gene in *G*. *thermoglucosidasius* (5’-TTAGTTTATATGATAGACAAAC-3’) shares 73% similarity with that of *B*. *subtilis*.

**Fig 4:**
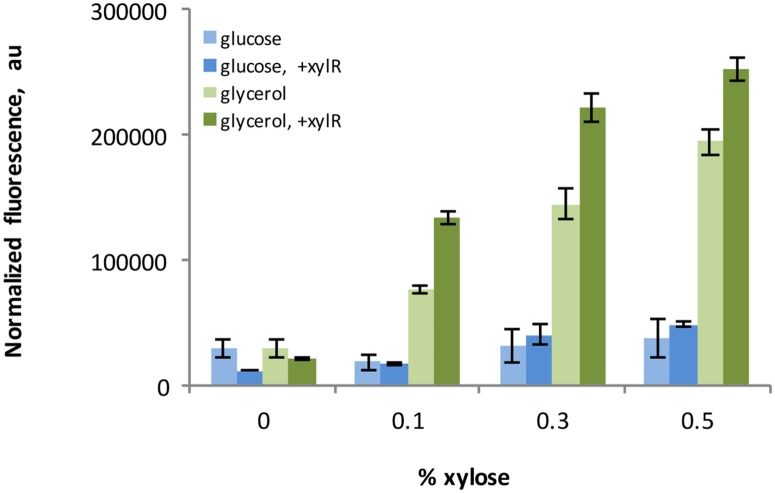
Inducible protein expression. Expression of Superfolder GFP is controlled by different concentrations of xylose, when its gene is expressed from the inducible promoter P_*xylA*_. The main carbon source was 0.5% (w/v) glucose or 0.5% (w/v) glycerol as indicated. Constructs further carrying the regulator gene *xylR* on the same plasmid are depicted by dark color.

The promoter from the *G*. *thermoglucosidasius* C56-YS93 xylose isomerase (*xylA*, locus tag Geoth_2243) was examined by fusing a 160 bp region immediately upstream from the *xylA* gene to a gene encoding sfGFP on a plasmid. The expression of sfGFP was measured for cells exposed to a range of xylose concentrations from 0 to 0.5% (w/v) with either 0.5% (w/v) glucose or 0.5% (w/v) glycerol as a main carbon source. For the glycerol medium, a step-wise increase in sfGFP expression was observed as a function of increasing xylose concentration, while the level of induction was less pronounced when glucose was present in the medium (Fig 4). The dynamic range of expression also varied significantly, where 2-fold difference was observed in glucose medium compared to 6.5-fold when cells were grown on glycerol medium. The basal expression from the non-induced promoter in glucose medium was lower when compared to the one with glycerol.

A considerable basal expression from the uninduced P_*xylA*_ was observed for both carbon sources. We hypothesized that it might be due to a repressor protein being titrated out by multiple copies of the extrachromosomal P_*xylA*_-sfGFP construct. Based on the homology of *xylA* and its operator to those in *B*. *subtilis* [45], the regulation mechanism of *xylA* expression may likely be similar in *G*. *thermoglucosidasius* as it is in *B*. *subtilis*, where XylR is a repressor of *xylA* gene expression [44]. Hence, in order to make a tighter promoter system, we expressed a putative *xylR* gene (Geoth_1256) with its native promoter and terminator on the same plasmid. This resulted in a decrease in basal sfGFP expression, although some expression still remained (Fig 4). At zero or low inducer concentrations, additional copies of *xylR* decreased sfGFP expression. However, the effect was reversed at higher concentrations (Fig 4). Under these conditions overexpression of XylR surprisingly resulted in higher expression from P_*xylA*_. The sfGFP expression levels in pIP26 (*xylR* + P_*xylA*_::sfGFP) differed almost 12-fold between uninduced and fully induced conditions when cells were grown in medium containing glycerol as a carbon source.

### Xylanase production using the P_*xylA*_ expression system

Many *Geobacillus* species possess a conserved cluster of about 200 kb within a genome containing the genes for xylan utilization, notably a number of xylanases [19]. Xylanases are widely used in paper mill industry, animal feed processing and bakery, and the use of thermostable enzymes is also advantageous in certain fields. Therefore, we sought to use the strain and tools characterized above to overexpress two enzymes: a endo-1,4-β-xylanase native to *G*. *thermoglucosidasius* C56-YS93 (locus tag Geoth_2264, RefSeq GEOTH_RS11140) and the xylanase T-6 encoded by *xynA* in *G*. *thermodenitrificans* NG80-2 (locus tag GTNG_1761, RefSeq GTNG_RS09220) as relevant models for homologous and heterologous protein expression. In addition, xylanase T-6 has a putative N-terminal 28-amino acid signal peptide (MLKRSRKAIIVGFSFMLLLPLGMTNALA) predicted by SignalP 4.1 server [47] that potentially enables it to be secreted from the cell. Endo-1,4-β-xylanase (Geoth_2264) lacks a signal peptide.

**Fig 5:**
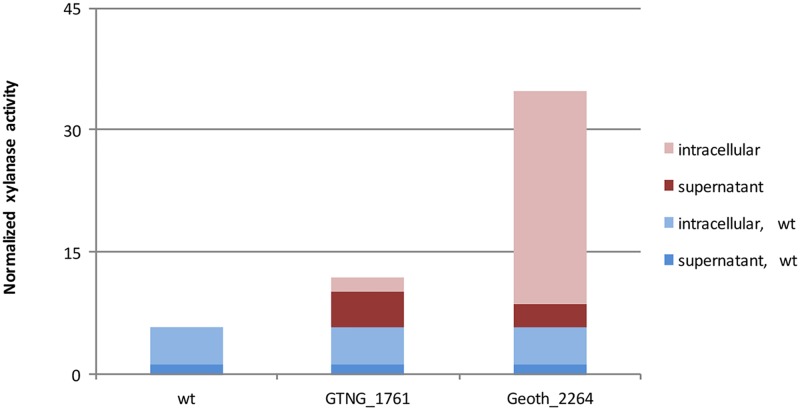
Application of induction system for expression of xylanases. Heterologous (GTNG_1761) and homologous (Geoth_2264) proteins overexpression in *G*. *thermoglucosidasius* under control of the P_*xylA*_ promoter. Basal xylanase activity in wild type strain is shown in blue, activities of overexpressed enzymes in red; dark and light colors correspond to extracellular and intracellular activities, respectively.

To demonstrate the applicability of the inducible P_*xylA*_ for protein expression, two xylanases were put under control of this promoter and expressed in the presence of the inducer (xylose). As shown in Fig 5, most of the xylanase T-6 activity (70%) was observed in the supernatant, indicating that it was secreted from the cell. Thus, in this case, a signal peptide from one species (*G*. *thermodenitrificans* NG80-2) was active in the other (*G*. *thermoglucosidasius* C56-YS93). The endo-xylanase was also successfully overexpressed and showed relatively high intracellular activity.

## Discussion

In this study we generated a set of tools for gene expression in *G*. *thermoglucosidasius* and characterized their use for homologous and heterologous protein production.

A library of ribosome binding sites was developed using the RBS Calculator [41]. It was previously shown that a computational model based on the thermodynamics of RNA binding to ribosome does not always accurately predict the actual translation efficiency [48]. Factors other than the strength of the Shine-Dalgarno sequence might play a role [49]; [50]. In this study the sfGFP expression levels generally correlated with predicted translation initiation rates, except for one outlier. One of the factors that may have influenced the accuracy of prediction in this study was the default settings of the RBS Calculator v1.1. In this version the default temperature is 37°C and could not be adjusted for growth optimum of 60°C of the thermophilic *G*. *thermodenitrificans* NG80-2, which was used as a model. Although the number of RBS sequences tested (six) may be too low to make general statements on predictability of the RBS Calculator for *Geobacillus* species, the library is large enough for practical purposes of controlling gene expression, as it covers a relatively wide range of translation efficiencies. Future research may use the RBS’ designed here in the context of the bicistronic architecture [51] to improve precision of protein biosynthesis, especially in cases of difficult-to-express proteins.

An inducible promoter of the *xylA* gene studied here showed a 12-fold dynamic range between uninduced and fully induced states, while at the same time demonstrating a significant basal activity. It is desirable for an inducible promoter to be tightly regulated, which means that it should have very low level of expression when not induced. In *B*. *subtilis*, the active repressor protein (XylR) binds to its motif in the promoter region. Additionally, the *xylA* gene is also negatively regulated by catabolite repression by CcpA in the presence of glucose-6-phosphate, where the cis acting element is the catabolite responsive element (CRE), a 14-bp sequence within the upper part of *xylA* [52]. In addition, glucose-6-phosphate can act on the activity of XylR itself [52]. However, CRE is absent in *xylA* gene in *G*. *thermoglucosidasius*, although the gene product is highly homologous (75% amino acid identity) to that of XylA from *B*. *subtilis*. Therefore, we could not use CRE to decrease the leakiness of *xylA* promoter (e.g. by fusing it to the heterologous gene). Importing the catabolite repression system from *B*. *subtilis* is hindered by its possibly lower thermostability. A homologue of the *B*. *subtilis ccpA* gene is present in *G*. *thermoglucosidasius* C56-YS93 genome (Geoth_0851). However, to the best of our knowledge, its target sequence is currently unknown.

Additional copies of the putative *xylR* gene did on the other hand reduce basal expression from P_*xylA*_. The repression by XylR was significantly more pronounced in the presence of glucose, which is in agreement with *B*. *subtilis* model. However, at higher concentrations of the inducer xylose, the presence of additional XylR resulted in increased P_*xylA*_ activity, i.e. its repressor activity was reversed. This may be due to an unknown mechanism of XylR-mediated regulation in *Geobacillus* spp., so that at zero or low xylose concentrations XylR acts as a repressor, while at high concentrations in becomes an activator. Similar cases of such dual repressors/activators are known in some bacteria, as for example the Cra protein [53] and AraC regulator [54] in *E*. *coli*.

One possible way to decrease basal expression from an inducible promoter is to subject it to directed evolution. It involves applying error-prone PCR to a parent promoter in order to generate a library of promoters with random mutations. This library can then be screened for desirable properties. Apart from tighter promoter versions, a number of other useful properties could be searched for. These could include wider dynamic ranges, sensitivity (the rate at which induction increases with inducer), etc. [55].

Another possible candidate for an inducible system is the promoter of *araD* gene. AraD is a part of arabinose utilization system and in *B*. *subtilis* its expression and the expression of other genes in the same operon is induced by arabinose. It is controlled by the regulation protein AraR which binds to the operator sequence and acts as a repressor [56]. In the presence of arabinose it releases from DNA which makes the transcription possible. The arabinose utilization operons with regulatory and structural genes including *araR* and *araD* were characterized in at least one species of *Geobacillus* [57]. We also found that putative *araD* with a respective operator sequence (5’-ATTGTACGTACAA-3’) and *araR* are present in *G*. *thermodenitrificans* NG80-2 and *G*. *kaustophilus* HTA426. Future work will be needed to characterize this and other inducible promoter systems in *Geobacillus* strains.

Apart from the inducible *xylA* promoter, a library of 17 constitutive promoters was created and quantified in this study. Importantly, the dynamic range of the inducible P_*xylA*_ falls within the expression range of the library. This feature might find an application e.g. in cases where it is necessary to find an optimal expression level of a certain gene. It might be carried out by varying the activity of the inducible promoter, and afterwards placing the respective gene under the constitutive promoter of comparable strength.

This study provides a toolkit for controlled gene expression in *G*. *thermoglucosidasius*. Since there is a growing interest in *Geobacillus* spp. in both academia and industry, these tools would be valuable instruments for a number of different applications.

## References

[1] Saiki RK, Gelfand DH, Stoffel S, Scharf SJ, Higuchi R, Horn GT, et al Primer-directed enzymatic amplification of DNA with a thermostable DNA polymerase. Science. 1988;239: 487–491. 2448875 10.1126/science.2448875

[2] Sarrouh B, Santos TM, Miyoshi A, Dias R, Azevedo V. Up-to-date insight on industrial enzymes applications and global market. J Bioprocess Biotech. 2012;S4: 1–10.

[3] Haki GD, Rakshit SK. Developments in industrially important thermostable enzymes: A review. Bioresour Technol. 2003;89: 17–34. 12676497 10.1016/s0960-8524(03)00033-6

[4] Nazina TN, Tourova TP, Poltaraus AB, Novikova E V, Grigoryan AA, Ivanova AE, et al Taxonomic study of aerobic thermophilic bacilli: descriptions of Geobacillus subterraneus gen. nov., sp. nov. and Geobacillus uzenensis sp. nov. from petroleum reservoirs and transfer of Bacillus stearothermophilus, Bacillus thermo- catenulatus, Bacillus. Int J Syst Evol Microbiol. 2001;51: 433–446. 10.1099/00207713-51-2-433 11321089

[5] Chen XG, Stabnikova O, Tay JH, Wang JY, Tay STL. Thermoactive extracellular proteases of Geobacillus caldoproteolyticus, sp. nov., from sewage sludge. Extremophiles. 2004;8: 489–498. 10.1007/s00792-004-0412-5 15322950

[6] Mok SC, Teh AH, Saito JA, Najimudin N, Alam M. Crystal structure of a compact ??-amylase from Geobacillus thermoleovorans. Enzyme Microb Technol. Elsevier Inc.; 2013;53: 46–54. 10.1016/j.enzmictec.2013.03.009 23683704

[7] Leow TC, Rahman RNZRA, Basri M, Salleh AB. A thermoalkaliphilic lipase of Geobacillus sp. T1. Extremophiles. 2007;11: 527–535. 10.1007/s00792-007-0069-y 17426920

[8] Mechaly A, Teplitsky A, Belakhov V, Baasov T, Shoham G, Shoham Y. Overproduction and characterization of seleno-methionine xylanase T-6. J Biotechnol. 2000;78: 83–86. 10702913 10.1016/s0168-1656(99)00226-6

[9] Bosma EF, Van Der Oost J, De Vos WM, Van Kranenburg R. Sustainable production of bio-based chemicals by extremophiles. Curr Biotechnol. 2013;2: 360–379.

[10] Taylor MP, Eley KL, Martin S, Tuffin MI, Burton SG, Cowan DA. Thermophilic ethanologenesis: future prospects for second-generation bioethanol production. Trends Biotechnol. 2009;27: 398–405. 10.1016/j.tibtech.2009.03.006 19481826

[11] Wittlich P, Themann A, Vorlop KD. Conversion of glycerol to 1,3-propanediol by a newly isolated thermophilic strain. Biotechnol Lett. 2001;23: 463–466.

[12] Chung D, Cha M, Guss AM, Westpheling J. Direct conversion of plant biomass to ethanol by engineered Caldicellulosiruptor bescii. Proc Natl Acad Sci U S A. 2014;111: 8931–6. 10.1073/pnas.1402210111 24889625 PMC4066518

[13] Clark ME, Batty JD, van Buuren CB, Dew DW, Eamon MA. Biotechnology in minerals processing: Technological breakthroughs creating value. Hydrometallurgy. 2006;83: 3–9.

[14] Cripps RE, Eley K, Leak DJ, Rudd B, Taylor M, Todd M, et al Metabolic engineering of Geobacillus thermoglucosidasius for high yield ethanol production. Metab Eng. Elsevier; 2009;11: 398–408. 10.1016/j.ymben.2009.08.005 19703579

[15] Van Zyl LJ, Taylor MP, Eley K, Tuffin M, Cowan DA. Engineering pyruvate decarboxylase-mediated ethanol production in the thermophilic host Geobacillus thermoglucosidasius. Appl Microbiol Biot. 2014;98: 1247–1259. 10.1007/s00253-013-5380-1 24276622

[16] Lin PP, Rabe KS, Takasumi JL, Kadisch M, Arnold FH, Liao JC. Isobutanol production at elevated temperatures in thermophilic Geobacillus thermoglucosidasius. Metab Eng. Elsevier; 2014;24: 1–8. 10.1016/j.ymben.2014.03.006 24721011

[17] Suzuki H, Yoshida KI, Ohshima T. Polysaccharide-degrading thermophiles generated by heterologous gene expression in Geobacillus kaustophilus HTA426. Appl Env Microb. 2013;79: 5151–5158. 10.1128/AEM.01506-13 PMC3753961 23793634

[18] Kananavičiute R, Čitavičius D. Genetic engineering of Geobacillus spp. J Microbiol Methods. 2015;111: 31–39. 10.1016/j.mimet.2015.02.002 25659824

[19] Brumm PJ, De Maayer P, Mead DA, Cowan DA. Genomic analysis of six new Geobacillus strains reveals highly conserved carbohydrate degradation architectures and strategies. Front Microbiol. 2015;6: 1–15. 26029180 10.3389/fmicb.2015.00430 PMC4428132

[20] Martin C, Puls J, Saake B, Schreiber a. Effect of Glycerol Preatreatment on Component Recovery and Enzymatic Hydrolysis of Sugarcane Bagasse. Cellul Chem Technol. 2011;45: 487–494. Available: <Go to ISI>://WOS:000299143700008

[21] Rastogi G, Bhalla A, Adhikari A, Bischoff KM, Hughes SR, Christopher LP, et al Characterization of thermostable cellulases produced by Bacillus and Geobacillus strains. Bioresour Technol. Elsevier Ltd; 2010;101: 8798–8806. 10.1016/j.biortech.2010.06.001 20599378

[22] Imanaka T, Fujii M, Aramori I, Aiba S. Transformation of Bacillus stearothermophilus with plasmid DNA and characterization of shuttle vector plasmids between Bacillus stearothermophilus and Bacillus subtilis. J Bacteriol. 1982;149: 824–830. 6277855 10.1128/jb.149.3.824-830.1982 PMC216468

[23] Narumi I, Sawakami K, Nakamoto S, Nakayama N, Yanagisawa T, Takahashi N, et al A newly isolated Bacillus stearotheromophilus K1041 and its transformation by electroporation. Biotechnol Tech. 1992;6: 83–86.

[24] Zeigler DR. The genus Geobacillus. Bacillus genetic stock center catalog of strains Seventh Edition. 2001. http://scholar.google.com/scholar?hl=en&btnG=Search&q=intitle:Catalog+of+Strains#4

[25] Taylor MP, Esteban CD, Leak DJ. Development of a versatile shuttle vector for gene expression in Geobacillus spp. Plasmid. 2008;60: 45–52. 10.1016/j.plasmid.2008.04.001 18501964

[26] Liao H, McKenzie T, Hageman R. Isolation of a thermostable enzyme variant by cloning and selection in a thermophile. Proc Natl Acad Sci U S A. 1986;83: 576–80. 3003740 10.1073/pnas.83.3.576 PMC322906

[27] Blanchard K, Robic S, Matsumura I. Transformable facultative thermophile Geobacillus stearothermophilus NUB3621 as a host strain for metabolic engineering. Appl Microbiol Biot. 2014;98: 6715–6723. 10.1007/s00253-014-5746-z PMC4251812 24788326

[28] Ingram LO, Jarboe LR, Zhang X, Wang X, Moore JC, Shanmugam KT. Metabolic engineering for production of biorenewable fuels and chemicals: Contributions of synthetic biology. J Biomed Biotechnol. 2010;2010. 10.1155/2010/761042 PMC2857869 20414363

[29] Makrides SC, Makrides SC. Strategies for Achieving High-Level Expression of Genes in Escherichia coli. Microbiol Rev. 1996;60: 512–538. 8840785 10.1128/mr.60.3.512-538.1996 PMC239455

[30] Sambrook J, Fritsch EF, Maniatis T. Molecular cloning: a laboratory manual. Cold Spring Harbor Laboratory Press 1989 p. 626.

[31] Fong JCN, Svenson CJ, Nakasugi K, Leong CTC, Bowman JP, Chen B, et al Isolation and characterization of two novel ethanol-tolerant facultative-anaerobic thermophilic bacteria strains from waste compost. Extremophiles. 2006;10: 363–372. 10.1007/s00792-006-0507-2 16532362

[32] Brumm P, Land ML, Hauser LJ, Jeffries CD, Chang Y-J, Mead DA. Complete genome sequences of Geobacillus thermoglucosidasius C56-YS93, a novel biomass degrader isolated from obsidian hot spring in Yellowstone National Park. Stand Genomic Sci. Standards in Genomic Sciences; 2015;10: 73 10.1186/s40793-015-0031-z 26442136 PMC4593210

[33] Cavaleiro AM, Kim SH, Seppälä S, Nielsen MT, Nørholm MHH. Accurate DNA Assembly and Genome Engineering with Optimized Uracil Excision Cloning. ACS Synth Biol. 2015;4: 1042–1046. 10.1021/acssynbio.5b00113 26263045

[34] Pédelacq J-D, Cabantous S, Tran T, Terwilliger TC, Waldo GS. Engineering and characterization of a superfolder green fluorescent protein. Nat Biotechnol. 2006;24: 79–88. 10.1038/nbt1172 16369541

[35] Couñago R, Chen S, Shamoo Y. In Vivo Molecular Evolution Reveals Biophysical Origins of Organismal Fitness. Mol Cell. 2006;22: 441–449. 10.1016/j.molcel.2006.04.012 16713575

[36] Jensen PR, Hammer K. The sequence of spacers between the consensus sequences modulates the strength of prokaryotic promoters. Appl Env Microbiol. 1998;64: 82–87. 9435063 10.1128/aem.64.1.82-87.1998 PMC124675

[37] Braatsch S, Helmark S, Kranz H, Koebmann B, Jensen PR. Rapid fine tuning of Eschericia coli gene expression. Biotechniques. 2008;45: 1–4. 10.2144/000112907 18778259

[38] Nevoigt E, Kohnke J, Fischer CR, Alper H, Stahl U, Stephanopoulos G. Engineering of promoter replacement cassettes for fine-tuning of gene expression in Saccharomyces cerevisiae. Appl Environ Microbiol. 2006;72: 5266–5273. 10.1128/AEM.00530-06 16885275 PMC1538763

[39] Schon U, Schumann W. Molecular cloning, sequencing, and transcriptional analysis of the groESL operon from Bacillus stearothermophilus. J Bacteriol. 1993;175: 2465–2469. 8096841 10.1128/jb.175.8.2465-2469.1993 PMC204540

[40] Zuber U, Schumann W. CIRCE, a novel heat-shock element involved in regulation of heat-shock operon dnaK of Bacillus subtilis. J Bacteriol. 1994;176: 1359–1363. Available: <Go to ISI>://A1994MY35100020 8113175 10.1128/jb.176.5.1359-1363.1994 PMC205200

[41] Salis HM, Mirsky EA, Voigt CA. Automated design of synthetic ribosome binding sites to control protein expression. Nat Biotechnol. 2009;27: 946–950. 10.1038/nbt.1568 19801975 PMC2782888

[42] Espah Borujeni A, Channarasappa AS, Salis HM. Translation rate is controlled by coupled trade-offs between site accessibility, selective RNA unfolding and sliding at upstream standby sites. Nucleic Acids Res. 2014;42: 2646–2659. 10.1093/nar/gkt1139 24234441 PMC3936740

[43] Bonde MT, Pedersen M, Klausen MS, Jensen SI, Wulff T, Harrison S, et al Predictable tuning of protein expression in bacteria. Nat Methods. 2016;13: 233–236. 10.1038/nmeth.3727 26752768

[44] Gartner D, Degenkolb J, Ripperger JA, Allmansberger R, Hillen W. Regulation of the Bacillus subtilis W23 xylose utilization operon: interaction of the Xyl repressor with the xyl operator and the inducer xylose. Mol Gen Genet. 1992;232: 415–422. Available: http://www.ncbi.nlm.nih.gov/pubmed/1588910 1588910 10.1007/BF00266245

[45] Gu Y, Ding Y, Ren C, Sun Z, Rodionov Da, Zhang W, et al Reconstruction of xylose utilization pathway and regulons in Firmicutes. BMC Genomics. 2010;11: 255 10.1186/1471-2164-11-255 20406496 PMC2873477

[46] Stammen S, Müller BK, Korneli C, Biedendieck R, Gamer M, Franco-Lara E, et al High-yield intra- And extracellular protein production using bacillus megaterium. Appl Environ Microbiol. 2010;76: 4037–4046. 10.1128/AEM.00431-10 20435764 PMC2893505

[47] Petersen TN, Brunak S, von Heijne G, Nielsen H. SignalP 4.0: discriminating signal peptides from transmembrane regions. Nat Methods. Nature Publishing Group; 2011;8: 785–786. 10.1038/nmeth.1701 21959131

[48] Li GW, Burkhardt D, Gross C, Weissman JS. Quantifying absolute protein synthesis rates reveals principles underlying allocation of cellular resources. Cell. Elsevier; 2014;157: 624–635. 10.1016/j.cell.2014.02.033 PMC4006352 24766808

[49] Nomura M, Gourse R, Baughman G. Regulation of the synthesis of ribosomes and ribosomal components. Annu Rev Biochem. 1984;53: 75–117. 10.1146/annurev.bi.53.070184.000451 6206783

[50] Mccarthy JEG, Gualerzl C. Translational control of prokaryotic gene expression. Trends Genet. 1990;6: 78–85. 2183416 10.1016/0168-9525(90)90098-q

[51] Mutalik VK, Guimaraes JC, Cambray G, Lam C, Christoffersen MJ, Mai Q-A, et al Precise and reliable gene expression via standard transcription and translation initiation elements. Nat Methods. 2013;10: 354–360. 10.1038/nmeth.2404 23474465

[52] Kraus A, Hueck C, Gartner D, Hillen W. Catabolite repression of the Bacillus subtilis xyl operon involves a cis element functional in the context of an unrelated sequence, and glucose exerts additional xylR-dependent repression. J Bacteriol. 1994;176: 1738–1745. 8132469 10.1128/jb.176.6.1738-1745.1994 PMC205262

[53] Saier MH, Ramseier TM. The Catabolite Repressor / Activator (Cra) Protein of Enteric Bacteria. Microbiology. 1996;178: 3411–3417. Available: http://view.ncbi.nlm.nih.gov/pubmed/8655535 10.1128/jb.178.12.3411-3417.1996 PMC178107 8655535

[54] Martin RG, Rosner JL. The AraC transcriptional activators. Curr Opin Microbiol. 2001;4: 132–137. 11282467 10.1016/s1369-5274(00)00178-8

[55] Tyo KEJ, Nevoigt E, Stephanopoulos G. Directed evolution of promoters and tandem gene arrays for customizing RNA synthesis rates and regulation [Internet] 1st ed Methods in Enzymology. Elsevier Inc; 2011. 10.1016/B978-0-12-385075-1.00006-8 21601085

[56] Mota LJ, Tavares P, Sá-Noguelra I. Mode of action of AraR, the key regulator of L-arabinose metabolism in Bacillus subtilis. Mol Microbiol. 1999;33: 476–489. 10417639 10.1046/j.1365-2958.1999.01484.x

[57] Shulami S, Raz-Pasteur A, Tabachnikov O, Gilead-Gropper S, Shner I, Shoham Y. The L-arabinan utilization system of Geobacillus stearothermophilus. J Bacteriol. 2011;193: 2838–2850. 10.1128/JB.00222-11 21460081 PMC3133107

